# Replication Kinetics for a Reporter Merkel Cell Polyomavirus

**DOI:** 10.3390/v14030473

**Published:** 2022-02-25

**Authors:** Bizunesh Abere, Hongzhao Zhou, Masahiro Shuda, Donna B. Stolz, Kyle Rapchak, Patrick S. Moore, Yuan Chang

**Affiliations:** 1 Cancer Virology Program, Hillman Cancer Center, University of Pittsburgh, Pittsburgh, PA 15213, USA; baa103@pitt.edu (B.A.); hoz36@pitt.edu (H.Z.); mas253@pitt.edu (M.S.); ksr52@pitt.edu (K.R.); 2 Department of Microbiology and Molecular Genetics, University of Pittsburgh, Pittsburgh, PA 15213, USA; 3 Department Cell Biology, Center for Biologic Imaging, University of Pittsburgh, Pittsburgh, PA 15213, USA; donna.stolz@pitt.edu; 4 Department of Pathology, University of Pittsburgh, Pittsburgh, PA 15213, USA

**Keywords:** Merkel cell polyomavirus, minicircle, replication

## Abstract

Merkel cell polyomavirus (MCV) causes one of the most aggressive human skin cancers, but laboratory studies on MCV replication have proven technically difficult. We report the first recombinase-mediated MCV minicircle (MCVmc) system that generates high levels of circularized virus, allowing facile MCV genetic manipulation and characterization of viral gene expression kinetics during replication. Mutations to Fbw7, Skp2, β-TrCP and hVam6p interaction sites, or to the stem loop sequence for the MCV-encoded miRNA precursor, markedly increase viral replication, whereas point mutation to an origin-binding site eliminates active virus replication. To further increase the utility of this system, an mScarlet fusion protein was inserted into the VP1 c-terminus to generate a non-infectious reporter virus for studies on virus kinetics. When this reporter virus genome is heterologously expressed together with MCV VP1 and VP2, virus-like particles are generated. The reporter virus genome is encapsidated and can be used at lower biosafety levels for one-round infection studies. Our findings reveal that MCV has multiple, self-encoded viral restriction mechanisms to promote viral latency over lytic replication, and these mechanisms are now amenable to examination using a recombinase technology.

## 1. Introduction

Merkel cell polyomavirus (MCV) causes most cases of Merkel cell carcinoma (MCC), an uncommon human skin cancer associated with immunosuppression [1] and aging [2]. MCC is highly aggressive with a 54% 5-year survival rate [3]. MCV is a near-ubiquitous component of viral skin flora; it causes MCC if the viral genome becomes integrated into the host genome and acquires mutations ablating replication functions [4,5]. Unlike virus-positive MCC, virus-negative MCC shows high levels of UV-induced somatic mutagenesis that phenocopy MCV oncoprotein functions [6,7,8,9,10].

MCV is a nonenveloped, double-stranded DNA virus with a 5.4 kb genome partitioned into early (ER) and late regions (LR) by a non-coding control region (NCCR) [4,11]. In addition to the replication origin, the NCCR contains promoter elements regulating early and late gene expression [12]. The ER encodes several proteins designated tumor antigens (T-Ags), such as large T-Ag (LT), small T-Ag (sT), 57kT [5] and alternative LT open reading frame (ALTO) [13]. The ER also expresses an auto-repressive miRNA [14,15] as well as circular RNAs that antagonize miRNA activity [16] and may also express alternative T peptides through cap-independent translation [17]. The LR expresses at least two viral structure proteins, VP1 and VP2, which comprise the viral capsid [18]. Despite the simplicity of its two-gene genome (ER and LR), actual MCV gene expression and replication is complex due to alternative splicing, alternative translation initiation, promoter regulation, proteostatic regulation and miRNA–circRNA feedback loops [5,12,13,16].

MCV is widely detected by PCR on skin and by serologic assays in the blood of individuals world-wide, providing evidence for persistent life-long infection [19,20,21,22]. Despite this, direct visualization of the virus or viral molecules in non-neoplastic human skin has not been achieved, presumably due to low copy number and viral latency. Furthermore, studies on the MCV life cycle have been hampered by an inability to achieve permissive replication and infection in tissue culture [18,23,24,25,26]. Recombinant MCV genomes can be circularized and transfected into producer cells to harvest viruses for infection studies in human dermal fibroblasts [27], but this approach is technically complex.

The replication lifecycle of MCV is similar, but not identical to replication in other polyomaviruses [23,24,28]. Based on in vitro virus generation and virus-like particle (VLP) studies, MCV capsids bind to heparin sulfate moieties on host cell surfaces followed by secondary interaction with sialylated glycans [25,29]. MCV utilizes caveolin/lipid raft-mediated endocytosis to transit the host cell plasma membrane but only minor populations of the internalized MCV virions are able to reach the endoplasmic reticulum, which has been suggested to be a bottleneck step during MCV infection [30,31]. It is not clear how MCV traffics from the endoplasmic reticulum to the nucleus.

After the MCV virion unpacks its genome in the nucleus, T-Ags are expressed that facilitate genome replication. LT is the only viral protein essential for MCV DNA replication [11,23]. It binds as a multimeric complex, presumably in a head-to-head double hexameric configuration, to GAGGC-like pentanucleotide sequences in the NCCR at the origin of replication (MCVori) and serves as an ATP-dependent DNA helicase [11]. Similar to SV40, formation of the replication initiation complex is presumed to involve the co-operative interaction of MCVori-bound LT, cellular DNA polymerase alpha/primase and replication protein A (RPA) [32,33,34,35]. While LT is absolutely required for replication, MCV sT is not, but it nevertheless serves as an accessory protein to enhance MCV replication [11,23]. By interacting with the SCF E3 ligase Fbw7 complex via its LT stabilization domain (LSD), sT inhibits Fbw7-related ubiquitin-mediated degradation of LT to markedly increase LT protein and MCV replication [36]. The contributions of 57kT and ALTO to MCV replication remain unknown.

Similar to other polyomaviruses [37,38], MCV LT appears to also exhibits transcriptional activity to auto-repress early gene transcription possibly by binding to as yet unmapped regions in the NCCR [12]. This places a natural transcriptional brake on MCV replication to inhibit the full lytic viral lifecycle. Multiple cellular factors are also known to interact with LT to restrict MCV genome replication. E3 ligases Skp2, β-TrCP and Fbw7 interact with specific phosphorylated residues to promote LT ubiquitylation and proteasomal degradation [12]. The evolutionarily conserved inhibition of MCV replication by degradation of preformed MCV LT is described as proteostatic viral latency [39]. Other restriction factors that affect LT’s replication activity include interaction with the deubiquitnase ubiquitin-specific protein 7 (Usp7) [40] and the vacuolar sorting protein hVam6p (Vps39) [23,41]. While Skp2-LT interactions may be shared with other polyomaviruses [39], it is largely unknown whether MCV LT-specific restriction factors are applicable to other polyomaviruses.

In addition to protein restriction pathways, MCV also encodes its own microRNA (miR-M1) from the negative strand of its ER [14,15,42]. MCV miR-M1 restrains MCV genomic replication by degrading LT antigen mRNA. A positive-strand circular RNA produced by backsplicing from exon 2 of the LT transcript antagonizes miR-M1, to enhance MCV replication [16]. The finely tuned choreography and interplay of these cellular and viral repression/amplification factors is likely to determine whether the virus enters into lytic virion production or remains as a latent episome in cells. Although this repertoire of replication control circuits is likely to provide a sophisticated cell type- and cell environment-specific mechanism for the binary decision to replicate or remain latent, the actual measurement of this interplay is limited by the absence of a permissive replication model.

To overcome these challenges, we adapted a recombinase-mediated minicircle system [43] to produce an MCV minicircle (MCVmc), which dramatically increases total circularized genomic DNA yield (from micrograms to milligrams) on a bacterial culture per volume basis. We show that mutagenesis of specific LT and miR-M1 sites robustly increases virus production, including support of the Fbw7-affected residue at LT serine 293 that has been confirmed [44] but challenged as a functional site [45]. By introducing a fluorescent tag fused to the c-terminus of VP1, we can track MCV replication after transfection using flow cytometry and can generate a biosafe, one-round infection/replication model to measure viral replication kinetics.

## 2. Materials and Methods

### 2.1. Cells

BJ-hTert cells were established by retroviral transduction of Babe hTert-puro (pBabe-hTert-puro: a gift from Roderick J. O’Sullivan, Hillman Cancer Center, University of Pittsburgh, Pittsburgh, PA, USA) into primary BJ foreskin fibroblast (ATCC CRL-2522) and selected with 2 µg/mL puromycin. To establish 293 TRE-sTco cells, 293 (ATCC CLR-1573) were transduced with pLenti TRE MCV sT [46] and selected with 2 µg/mL puromycin. The 293, 293 TRE-sTco, BJ and BJ-hTert cells were maintained in Dulbecco’s Modified Eagle Medium (DMEM; Corning, Manassas, VA, USA) supplemented with 10% FBS, while HFF-1 (ATCC SCRC-1041) cells were maintained in DMEM supplemented with 15% FBS. Human primary bone marrow or adipose tissue-derived mesenchymal stem cells (MSC-bm and MSC-a) [47], kindly provided by Shou-Jiang Gao (Hillman Cancer Center, University of Pittsburgh, PA, USA), were maintained in MSC medium (MSCM; ScienCell Research Laboratories, Carlsbad, CA, USA).

### 2.2. Plasmids and Constructs

The construction of pJ-MCV-HF, pMC-MCV and pMC-MCV-hpko plasmids have been previously described [16,23]. The MCV minicircle plasmids generated by restriction enzyme digestion cloning are listed in Table 1. To generate pMC-MCV-VP1-mS, a fragment containing the c-terminus of VP1 fused to mScarlet and part of the pMC backbone was produced by overlapping PCR using primers VP1 (2440-2466) F, VP1-mScarlet R, VP1-mScarlet F and mScarlet-attBVec R and was subsequently cloned into the pMC-MCV plasmid using PacI and XmaI restriction sites. pMC-MCV-VP1-P2A-mS was produced by restriction digestion of a fragment containing the c-terminus of VP1 fused to P2A-mScarlet and part of the pMC backbone generated with a GeneArt™ Seamless Cloning and Assembly Kit (cat. no. A13288, Thermo Fisher, Carlsbad, CA, USA) using primers VP1-P2A_FW, VP1-P2A_RV, P2A-mScarlet_FW and P2A-mScarlet_RV and subsequent cloning into the pMC-MCV backbone using PacI and XmaI restriction sites. The list and sources of all constructs are shown in Table S1. Primers used for plasmid construction are shown in Table 2.

### 2.3. Recircularization of MCV Genome by In Vitro Ligation and Mini-Circle System

Recircularization of MCV-HF genome (GenBank accession number: JF813003) by in vitro ligation was previously described [23]. To produce the recombinant MCV clone, the MCV-HF genome was cloned into the pSMART-LC-Amp vector (cat. no. 400300-2, Lucigen) using an EcoRI site present only once in the MCV genome. Since the resulting clone pSMART MCV-HF has two EcoRV blunt cutter enzyme sites immediately outside the EcoRI sites, a double digest using EcoRI and EcoRV results in a blunt-end vector fragment and an MCV-HF fragment with EcoRI cohesive ends for self-recircularization. pSMART-MCV-HF digested with EcoRI and EcoRV was recircularized by T4 DNA ligase at a low concentration (2.5 ng/µL) to reduce the formation of MCV concatemers.

Recombinase-mediated MCV recircularization was recently reported [16]. In brief, MCV-HF sequences with or without mutations (see plasmids and constructs, Table S1) were cloned into the pMC.BESPX plasmid which contains AttB and AttP sites. Constructs were transformed into ZYCY10P3S2T (cat. no. MN900A-1; System Biosciences, Palo Alto, CA, USA) competent cells, a kind gift from Mart Ustav (University of Tartu, Tartu, Estonia) and Alison McBride (National Institute of Allergy and Infectious Diseases, Bethesda, MD, USA). Transformed bacteria were cultured overnight in Terrific Broth (cat. no. T0918, Sigma-Aldrich, St. Louis, MO, USA) to an OD600 of 4–6 and induced by using an equal volume of buffer (0.04% l-arabinose and 40 mM NaOH in LB). After 6 h of induction, bacteria were harvested and MCVmc DNA genomes were purified using a Maxi prep kit (cat. no. 740416, Macherey-Nagel, Düren, Germany).

### 2.4. MCVmc Transfection and Replication Assay

In 6-well plates, 293 cells were plated and transfected with 1 µg of MCV-ligated, MCVmc, MCVmc-hpko, MCVmc-Rep^–^, MCVmc-Skp2^–^, MCVmc-Fbw7^–^ using FuGENE (cat. no. E2311, Promega, Madison, WI, USA), following the manufacture’s protocol. After 2 or 4 days post transfection, cells were collected for protein and DNA extraction.

Total genomic DNA was isolated using DNAzol reagent (cat. no. 10503027; Thermo Fisher, Warrington, UK), according to the manufacturer’s instructions, and resuspended in 0.1× TE buffer (1 mM Tris·HCl pH 8.0, 0.01 mM EDTA pH 8.0). Then, 5 µg of total genomic DNA was digested overnight using DpnI and BamHI restriction enzymes to remove transfected DNA produced in bacteria and linearize the MCV genome consecutively, after which 5 ng of MCV or 50 ng of GAPDH-digested DNA was used for quantification by qPCR.

### 2.5. Quantitation of MCV Genome Copy by Real-Time PCR

Quantitative PCR (qPCR) was performed using PowerUp™ SYBR™ Green master mix (A25778, Thermo Fisher, Vilnius, Lithuania) together with primers MCV DNA Fw: 5′-AAAACACCCAAAAGGCAATG-3′ and MCV DNA Rev: 5′-GCAGAGACACTCTTGCCACA-3′ to quantify MCV genome copy numbers and GAPDH DNA Fw: 5′-TGTGTCCCTCAATATGGTCCTGTC-3′ and GAPDH Rev: 5′-ATGGTGGTGAAGACGCCAGT-3′ to amplify endogenous control GAPDH DNA. Thermal cycling was performed on a QuantStudio™ 3 Real-Time PCR machine. Threshold cycle (CT) values were used to calculate DNA replication levels and were normalized to GAPDH. MCV genomic DNA replication levels were calculated according to the ΔΔCT method.

### 2.6. Immunoblotting

Total protein was extracted by lysing cells in 1% SDS buffer (1% SDS, 10 mM Tris-HCl, pH 8.0, 1 mM EDTA, pH 8.0) and subsequent sonication at 20% Amp for 5 s four times on ice. Protein concentration was then quantified using the DC Protein Assay Kit (cat. no. 5000116, Bio-Rad, Hercules, CA, USA). Then, 100 µg of total protein was separated by SDS-PAGE and transferred onto a nitrocellulose membrane. Membranes were then incubated with primary mouse monoclonal antibody to MCV LT and 57 kT (CM2B4), MCV VP1 (CM9B2), MCV ALTO (CM7B1), MCV sT (CM5E1) or mScarlet (cat. no. 6g6-100; Chromotek, Planegg-Martinsried, Germany) followed by 1:10,000 dilution of IRD800 conjugated goat anti-mouse secondary antibody (cat. no. 926-32210; LI-COR Biotechnology, Lincoln, NE, USA) in combination with a 1:20,000 dilution of Rhodamine conjugated anti-Tubulin antibody (cat. no. 12004163; Bio-Rad, Hercules, CA, USA). Signals were detected on a ChemiDoc imaging system (Bio-Rad, Hercules, CA, USA).

### 2.7. Immunofluorescence Assay (IFA)

Five days after transfection, U2OS or 293 cells grown on coverslips were fixed with 4% paraformaldehyde in PBS for 15 min at room temperature (RT), permeabilized with 0.1% Triton X-100 in PBS for 15 min at RT and incubated in blocking solution (5% normal Goat serum: cat. no. 9023, Sigma-Aldrich, St. Louis, MO, USA) for 1 h at RT. Coverslips were then incubated with primary mouse antibody to MCV LT (CM2B4) or MCV VP1 (CM9B2) for 1 h at 37 °C in a humidified chamber and washed three times in 1× PBS for 5 min at RT followed by incubation with AF-488 conjugated goat anti-mouse secondary antibody (cat. no. 11006, Invitrogen) for 30 min at 37 °C. For LT-VP1 co-staining in infected cells, CM2B4 antibody was conjugated with AF-488 using Alexa Fluor 488 antibody labeling kit (cat. no. A20181, Invitrogen, Eugene, OR, USA) and coverslips were sequentially stained first with CM9B2 primary antibody for 1 h at 37 °C followed by AF-568 conjugated goat anti-mouse secondary antibody (cat. no. 11004, Invitrogen, Eugene, OR, USA) for 30 min at 37 °C and then with AF-488 conjugated CM2B4 primary antibody to LT for 1 h at 37 °C. All antibody incubations were performed in a humidified chamber in the dark, with three washes in between each incubation period. After the final wash, cells were stained with 300 nM of DAPI in 1× PBS for 5 min, washed three times and coverslips were mounted on a glass slide. Images were acquired using an Olympus AX70 microscope with a QImaging QIClick charge-coupled device (CCD) camera and Q-Capture Pro 7 software for qualitative anlysis or a Cytation5 Imaging reader and Gen5 image analysis software (BioTek, Santa Clara, CA, USA) for quantitative analysis.

### 2.8. Virion Production

All wild-type MCV genomes used in this study conform to the HF strain (GenBank accession no. JF813003) [23]. The following day, 293 TRE-sTco cells were seeded at a density of 5 × 10^6^ cells per 100 mm dish and transfected with 10 µg of MCVmc or MCV-ligated DNA using Lipofectamine 2000 (Invitrogen, Carlsbad, CA, USA). One day after transfection, cells were passaged to two T75 flasks and treated with 500 ng/mL Dox on day 3 to induce sTco expression. Transfected cells were expanded from two T75 flasks to two or three T175 flasks in the presence of Dox treatment and harvested 10 days after transfection for MCV virion isolation. Cells were collected in PBS containing 9.5 mM MgCl_2_, 25 mM ammonium sulfate, 0.5% TritonX, 0.1% Benzonase, 1 mM ATP and 0.1% ATP-dependent DNase and incubated overnight at 37 °C to degrade unpackaged virus genomes. Nuclear fraction was then isolated by the addition of 720 mM of NaCl into the lysate. To purify MCV virion, supernatant from the lysate was overlayed on a 2.1 mL discontinuous Opti-Prep (iodixanol) (cat. no. D1556, Sigma-Aldrich, St. Louis, MO, USA) gradient (0.7 mL of 27%, 0.7 mL of 33% and 0.7 mL of 39%) and subjected to ultracentrifuge at 226,354× *g* for 2.5 h at 16 °C in an AH-650 swing rotor (Sorvall). Thirteen fractions (~400 µL) were collected from the top of the ultracentrifuge tube using a pipette and the fractions were used for immunoblotting, qPCR and infection assay.

### 2.9. Electron Microscopy

Suspensions of viruses were adhered to glow-discharged (Cressington 108Auto) Formvar-coated copper grids for 10 min. Extra solution was wicked away, then the samples were negatively stained with 1% aqueous uranyl acetate and solution was immediately wicked away. Samples were imaged on a JEOL JEM 1400 Flash transmission electron microscope (JEOL, Peabody, MA, USA) fitted with a BIOSPR12 bottom mount AMT camera (Advanced Micrscopy Techniques, Danvers, MA, USA). Images were taken at 80 kV.

### 2.10. MCV Infection Assay

Fibroblast cells (BJ, BJ.hTert, HFF-1) and MSC cells (MSC-bm, MSC-a) at a density of 2 × 10^5^ cells per well of a 6-well plate were infected with 4 × 10^5^ genome copy equivalent of MCV-ligated or MCVmc virion per cell in infection media, F12/DMEM medium containing 20 ng/mL EGF (cat. no. 78006, Stem Cell Technologies, Vancouver, BC, Canada), 20 ng/mL bFGF (cat. no. 78003, Stem Cell Technologies, Vancouver, BC, Canada), 3 µM CHIR9901 (cat. no. S2924, Sellechem, Houston, TX, USA) and 0.025 mg/mL collagenase IV (cat. no. 17104019, Thermo Fisher, Waltham, MA, USA), and seeded in a 6-well plate. After 3 days of incubation with the infection media, FCS was added at a final concentration (volume/volume) of 20% and cells were incubated for another 2 days. After 5 days of infection, cells were seeded in a 48-well plate, fixed and processed for the following day, as described in the previous section. The remaining cells were passaged to 6-well plates and harvested after 10 days of infection for southern hybridization.

For single round infection with VLP-packaged reporter viruses, 293 TRE-sTco cells were seeded at a density of at 2.5 × 10^5^ cells per well in 12-well plates and infected with 2 × 10^3^ genome copy equivalent of VLP-packaged MCV virions per cell in DMEM with no FBS. After 24 h, FBS was added at a concentration of 10% (volume/volume) and 500 ng/mL Dox was added to the medium after 3 days of infection. Starting at day 4 post infection, medium with Dox was replenished every day and imaging was performed at day 10 post infection.

### 2.11. Southern Blot

MCV-ligated or MCVmc-infected cells harvested after 10 days of infection were suspended in genomic DNA lysis buffer (10 mM Tris, 25 mM EDTA, 0.5% SDS, 100 mM NaCl) containing 0.1 mg/mL proteinase K and incubated overnight at 37 °C. The lysate was treated with 0.1 mg/mL RNase A for 1 h at 37 °C and genomic DNA was extracted by phenol-chloroform and ethanol precipitation. Genomic DNA (3 µg) was then digested with DpnI and EcoRI overnight and subsequently electrophoresed in a 0.8% agarose TAE gel. The DNA in the agarose gel was depurinated in 0.25 N HCl, treated with denaturation buffer (0.5 M NaOH, 1.5 M NaCl) and capillary-transferred onto a Hybond-N^+^ Nylon Membrane (cat. no. RPN303B, GE Healthcare, Chicago, IL, USA) overnight with 10× SSC (1.5 M NaCl, 150 mM Sodium Citrate, pH 7.0). After UV crosslinking (Stratalinker, Stratagene, San Diego, CA, USA), the membrane was treated with prehybridization buffer (5× SSPE (diluted from 20× solution containing 3 M NaCl, 0.2 M sodium phosphate monobasic monohydrate, 0.2 M EDTA), 2% SDS, 1× Denhardts (diluted from 50× Denhardts, cat. no. 750018, Thermofisher, Waltham, MA, USA), 10% dextran sulfate, sonicated salmon sperm 10 µg/mL) for 4 h at 65 °C. A denatured, biotin-11dUTP-labeled MCV DNA probe generated with the Bio-prime Array CGH kit (cat. no. 45-0048, Invitrogen, Waltham, MA, USA) using a full-length MCV genome as template was then added to the prehybridization buffer and incubated overnight at 65 °C. After washing twice with 2× SSPE and once with 0.1× SSPE at 60 °C, membranes were incubated for 1 h with IR800 Dye-conjugated streptavidin (cat. no. 926-32230, Li-COR, Lincoln, NE, USA), and the hybridization signal was detected with a Li-COR Odyssey Infrared Imaging System (Li-COR, Lincoln, NE, USA).

### 2.12. MCV Kinetics Assay

For the MCV kinetics assay, 293 cells were plated and transfected with MCVmc, MCVmc, VP1-mS or MCVmc. VP1-P2A-mS with FuGENE was as described before. The medium was refreshed each day for 2 days and cells were collected from day 0 through day 10 post transfection for analysis by immunoblotting (see previous section) or flowcytometry.

### 2.13. Flow Cytometry

Harvested cells were fixed in 4% PFA on ice for 15 min, stored in PBS with 0.02% NaN_3_ at 4 °C protected from light and were directly used to quantitate mScarlet-positive cells by flow cytometry on a BD LSR Fortessa cell analyzer (BD Biosciences, San Jose, CA, USA).

### 2.14. MCV Packaging Assay

To assay for the packaging efficiency of MCV fluorescent reporter viruses, cell lysate from 293 TRE-sTco cells that were transfected and harvested as described in virion production were overlayed on 9 mL discontinuous gradient (3 mL of 27%, 3 mL of 33%, 2 mL of 39% and 1 mL of 60% iodixanol) and subjected to ultracentrifuge at 41,000 rpm for 6 h at 16 °C in SW 41 Ti swing-bucket rotor (Beckman, Indianapolis, IN, USA). Twelve fractions (1 mL each) were collected from the top of the tube using a pipette. Fractions were used to perform immunoblotting, qPCR (in the presence or absence of Benzonase treatment) and an infection assay.

To assay for the packaging efficiency of the pseudovirus system, 293 TRE-sTco cells were co-transfected with 18 µg MCV genome DNA or reporter plasmid pEGFP-N1, 15 µg pWM plasmid (expressing VP1) and 5 µg ph2m plasmid (expressing VP2) in T75 flasks. Three days after transfection, cells were harvested and virions were purified as described above.

## 3. Results

### 3.1. MCV Genome Recircularization by Site-Specific Recombination

**Figure 1:**
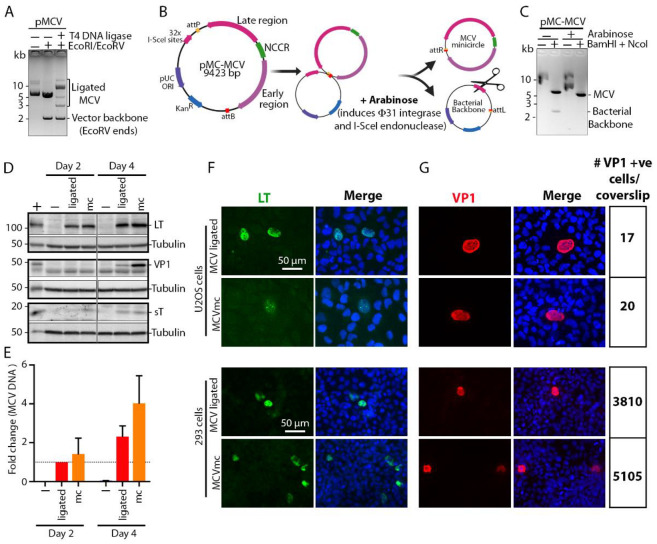
Development of a Merkel cell polyomavirus (MCV) molecular clone using minicircle (mc) technology. (**A**) Electrophoresis of MCV molecular clone re-circularized by T-4 DNA-ligase. DNA size markers are shown at the left of the gel image. (**B**) Schematic of recombinase-mediated re-circularization (minicircle technology) of MCV molecular clone (MCVmc). (**C**) Electrophoresis of MCV DNA extracted before and after recombination. DNA size markers are indicated at the left of the gel image. (**D**) Western blot of MCV-encoded proteins (LT, sT and VP1) from MCV-ligated and MCVmc-transfected 293 cells 2 and 4 days post transfection. Untransfected 293 cells were used as a negative control. The LT and VP1 expression constructs transfected in 293 cells were used as a positive control (indicated as +), while α-tubulin was used as an endogenous protein control. Protein molecular weight markers are shown on the left. The result is representative of three independent experiments. (**E**) Real-time PCR (qPCR) of the DpnI-resistant MCV genome from MCV-ligated or MCVmc-transfected 293 cells 2 and 4 days post transfection. Untransfected 293 cells were used as a negative control (indicated as −). The ΔΔCT method was used to determine MCV genome copy number fold change; GAPDH was used as an endogenous control, while the MCV-ligated 2 days post transfection group was used as the experimental control. Error bars indicate the ± SD of three independent experiments. (**F**–**G**) Immunofluorescence of LT-AF488 (pseudo color green), VP1-AF488 (pseudo color red) and DAPI (blue) in MCV-ligated and MCVmc-transfected 293 or U2OS cells 5 days post transfection. The numbers of VP1-positive cells per coverslip were counted with a Cytation 5 cell imaging multi-mode reader and are shown on the right. Images were originally acquired at 40× magnification.

We generated an MCV molecular clone using site-specific recombination, i.e., mini-circle (mc) technology [43] (Figure 1A-C) that has been used to produce HPV [48,49] and HBV [50] mc genomes. The MCV-HF genome (GenBank accession no. JF813003) [4] is linearized at nucleotide position 3146/3147 and cloned between the attB and attP sites in the pMC.BESPX vector to generate the parental pMC-MCV plasmid (Figure 1B). Transformation into *Escherishia coli* strain ZYCY10P3S2T allows arabinose-induction of bacteriophage ΦC31 integrase [43] that mediates recombination between the attB and attP sites, while I-SceI endonuclease, which is also induced by arabinose, digests the excised bacterial backbone containing 32 I-SceI recognition sites (Figure 1B). This produces covalently closed circular MCV minicircle (MCVmc) genomes, each with a 39 bp remnant scar sequence from the recombination (Figure 1B, C). Comparison of T4 DNA ligase (Figure 1A) and ΦC31 integrase (Figure 1C) recircularization shows that the minicircle system is not only more efficient than T4 DNA ligase-mediated re-circularization but also generates a single copy of the MCV genome, while ligation generates partial and multimeric forms, including forms with a vector backbone that might interfere with virus replication studies.

### 3.2. MCVmc Gene Expression

MCVmc and in vitro ligated MCV clones were compared for viral gene expression and replication capacity in 293 cells. With equal amounts of transfected genomic DNA, MCVmc expresses the LT-Ag protein at a comparable level to MCV-ligated DNA (Figure 1D). Expression of sT-Ag was slightly higher for MCVmc compared to MCV-ligated at two days post transfection. However, this difference in sT-Ag levels was minimal at 4 days post transfection (Figure 1D). The VP1 late gene product was detectable only at four days post transfection in both MCVmc and MCV-ligated transfected 293 cells. The level of VP1 was slightly higher in MCVmc transfected cells compared to MCV-ligated transfected cells (Figure 1D). Consistent with immunoblot results, MCVmc genomic DNA replicated at a higher rate compared to the MCV-ligated genome, as shown by qPCR of DpnI-resistant DNA (Figure 1E); comparable or higher expression for MCVmc was confirmed by immunofluorescence using antibodies to LT-Ag (Figure 1F) and VP1 (Figure 1G) on transfected 293 or U2OS cells, although transfection efficiency and viral gene expression was markedly lower for U2OS cells (Figure 1G). Notably, expression of VP1 protein, which serves as a surrogate marker for the complete cycle progression of DNA replication [51,52] in the MCVmc system, indicates that the “scar” of 39 additional non-MCV nucleotides extra sequences at the recombinase site does not interfere with MCV replication.

### 3.3. MCVmc In Vitro Transmission

**Figure 2:**
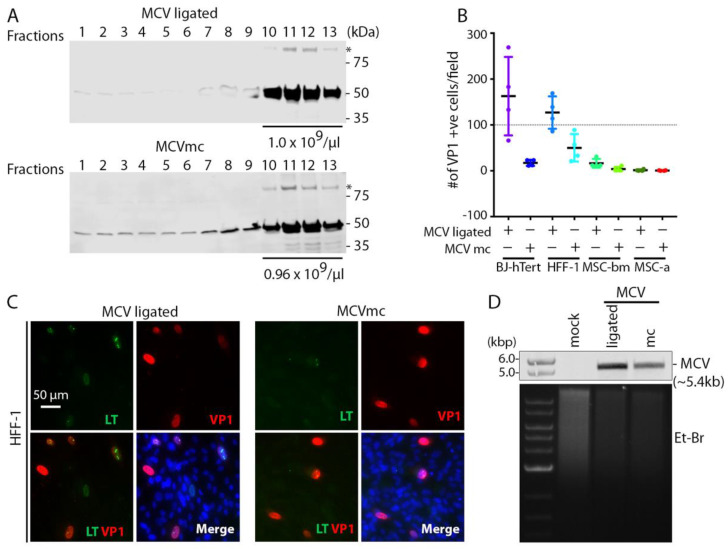
MCVmc is infectious in primary cells. (**A**) Western blot of MCV-ligated and MCVmc virions fractions from Opti-Prep (iodixanol) gradient purification. Fractions 1–13 (top to bottom) are indicated. * Indicates VP1 dimer. Fractions 10 through 13 were mixed and MCV genome copy numbers per µL are shown at the bottom of virion-positive fractions. Protein molecular weight markers are shown on the right. Results are representative of three independent experiments. (**B**) Quantification of the number of VP1-positive cells per 4× magnification field in BJ-hTert, HFF-1, MSC-bm and MSC-a cells infected with MCV-ligated or MCVmc virus from (**A**), 6 days post infection, by IFA of VP1. Four fields from one cover slip for each cell line was counted for MCVmc and MCV-ligated. (**C**) Representative immunofluorescence images showing LT-AF488 (pseudo color green), VP1-AF568 (pseudo color red), together with DAPI (blue) in MCV-ligated and MCVmc-infected HFF-1 cells 6 days post infection. Images were originally acquired at 40× magnification. Images (**C**) were acquired and the number of VP1 positive cells (**B**) was counted using a Cytation 5 cell imaging multi-mode reader. Results represent three independent experiments. (**D**) Southern blot of MCV genome from BJ-hTert cells infected with MCV-ligated or MCVmc. Mock-infected cells were used as a negative control. The relative amount of total DNA loaded is shown by EtBr staining and DNA size markers are shown in the left. Results represent three independent experiments.

sT is an important replication accessory protein that stabilizes and increases LT accumulation. To maximize virion production, we transfected MCVmc or MCV-ligated into 293 TRE-sTco cells with a stably integrated, doxycycline (Dox)-inducible, codon-optimized MCV sT cassette and purified cell lysates on OptiPrep (iodixanol) gradients (Figure 2A). VP1-positive fractions 10 through 13 were pooled and 1.0 × 10^10^ genome copy equivalent of viral particles were used to infect a panel of primary cell lines (Figure 2B–D and Figure S1). Human foreskin fibroblast (HFF-1), BJ-hTert, bone marrow-derived human mesenchymal stem cells (MSC-bm) and adipose-derived MSCs (MSC-a) show infection with both MCV-ligated and MCVmc. MCVmc virions had reduced infection compared to MCV-ligated as measured by an IFA of LT and VP1 expression (Figure 2B,C and Figure S1). This may be due to the incomplete digestion of unpackaged DNA that interferes with accurate MCV copy quantification or a difference in DNA backbones. This was confirmed by Southern blotting for MCV genome DNA in BJ-hTert cells (Figure 2D).

### 3.4. Mutagenesis of the MCVmc Genome and Cell-Specific Effects on Replication

**Figure 3:**
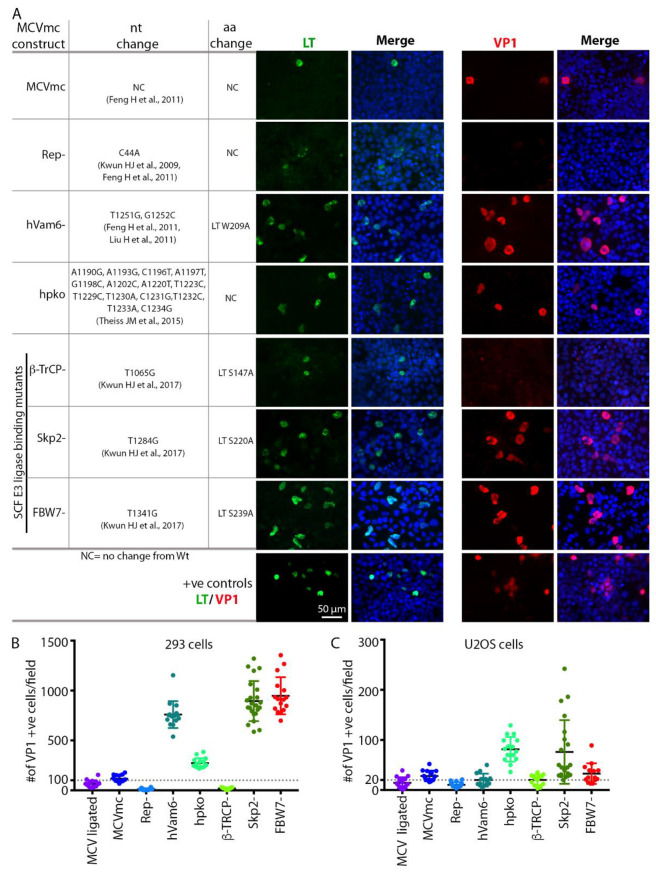
MCVmc is amenable to viral gene mutational analysis. (**A**) Left panel shows a table of tested MCV mutants; nucleotide (nt) and amino acid (aa) changes (based on HF strain: GenBank #JF813003) are noted for each mutant [11,12,15,23,41]. Right panel shows immunofluorescence of LT-AF488 (pseudo color green) or VP1-AF488 (pseudo color red) and DAPI (blue) in 293 cells transfected with MCVmc or mutants 5 days post transfection. LT or VP1-expression construct transfected 293 cells were used as positive controls. Images were originally acquired at 40× magnification. (**B**,**C**) Quantification of the number of VP1 positive cells per 4× magnification field in 293 or U2OS cells transfected with MCVmc or mutants 5 days post transfection. The number of VP1 positive cell was quantified using a Cytation 5 cell imaging multi-mode reader.

In a proof-of-principle experiment, previously reported mutations known to affect MCV replication were introduced into the parental pMC-MCV plasmid (Table 1). The MCVmc mutants generated include MCVmc-Rep^–^ which has a single nucleotide (C44A) mutation in the NCCR viral origin at a LT-binding pentanucleotide that abrogates viral genome replication [11,23]. Mutations to eliminate LT binding to hVam6p (MCV-hVam6p^–^; LT W209A) [23,41] and interactions with the Skip-Cul-Fbox (SCF) E3 ligases β-TrCp, Fbw7 and Skp2 [12] were also made. Additionally, we generated MCVmc.hpko, a virus with mutations to the hairpin loop required to produce miR-M1 [15] (Figure 3A).

Transfection of various mutated MCVmcs (Table 1) into either 293 or U2OS cells allowed both qualitative (Figure 3A and Figure S2) and quantitative (Figure 3B, C) assessment of replication permissivity as compared to wild-type MCVmcs. LT and VP1 positive cells displayed nuclei that are 2–5-fold larger than uninfected cells with condensed LT nuclear puncta consistent with viral replication centers, particularly in U2OS cells (Figure 3A and Figure S2). T4 DNA ligase-treated and MCVmc wild-type genomes showed similar low-level replication (measured by VP1 expression) that was, nevertheless, significantly greater than the MCVmc-Rep^–^ mutant. Mutation of the LT β-TrCP phosphodegron site (S147A) required for replication also did not show VP1 expression, indicative of replication loss, as previously reported [12]. Elimination of the Fbw7 interaction site markedly increased MCV replication in 293 cells (Figure 3B) but not U2OS cells, a pattern similar to that seen for the Vam6p binding site mutant while the Skp2 phosphodegron mutant increased MCV replication in both cell types. MCVmc carrying mutations in the miR-M1 hairpin markedly enhanced MCV replication independent of the cell type.

### 3.5. Generation of Fluorescent MCV Reporter Viruses (MCVmc.VP1-mS and MCVmc.VP1-P2A-mS)

**Figure 4:**
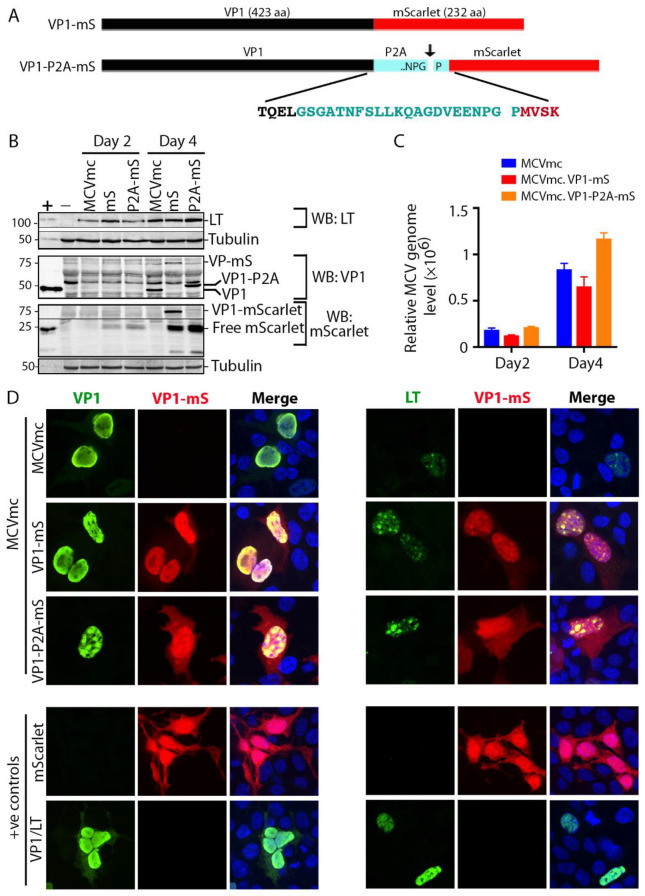
Generation of an mScarlet reporter MCVmc. (**A**) Schematic of VP1 fluorescent fusion constructs representing VP1 (black) containing mScarlet (red) tag in the presence (cyan) or absence of a P2A linker. Arrow indicates the P2A ribosome-skipping site and the whole P2A peptide sequence (cyan) is shown with four flanking amino acids from VP1 (black) and mScarlet (red). (**B**) Immunoblot of MCV-encoded proteins (LT and VP1) and mScarlet in 293 cells transfected with MCVmc, MCVmc.VP1-mS and MCVmc. VP1-P2A-mS after 2 and 4 days post transfection. Un-transfected 293 cells were used as a negative control (−), while 293 cells transfected with LT or VP1 expression construct were used as a positive control (+). α-tubulin was used as an endogenous protein-loading control. Protein molecular weight markers are shown on the left. (**C**) Quantification of DpnI-resistant replicated MCV DNA in 293 cells transfected with MCVmc, MCVmc.VP1-mS or MCVmc. VP1-P2A-mS 2 and 4 days post transfection qPCR results. The ΔΔCT method was used to calculate relative MCV DNA levels; GAPDH was used as the endogenous loading control, while MCVmc was used as the experimental control. Error bars indicate ± SD of three independent replicates. (**D**) Confocal images of mScarlet expression (pseudo color red) and LT-AF488 (pseudo color green) or VP1-AF488 (pseudo color Green) immunofluorescence in MCVmc, MCVmc.VP1-mS and MCVmc. VP1-P2A-mS-transfected 293 cells 5 days post transfection. LT, VP1 or mScarlet expression construct-transfected 293 cells were used as positive controls. Images were originally acquired at ×40 magnification.

To directly visualize the viral replication process in real time, we fused an mScarlet fluorescent protein coding sequence 3′ to the MCV VP1 protein-coding sequence (MCVmc.VP1-mS) to generate a VP1-mScarlet fusion protein (Figure 4A). In a second reporter virus (MCVmc.VP1-P2A-mS), the porcine teschovirus-1 2A peptide sequence (P2A: GSGATNFSLLKQAGDVEENPGP) that initiates ribosome skipping during translation [53] was engineered between VP1 and mScarlet proteins to minimize functional consequences of the 232 amino acid fusion tag. Nevertheless, MCVmc.VP1-P2A-mS still carries 21 extra non-MCV amino acids on the c-terminus of VP1.

293 cells were transfected with these MCVmc reporter genomes and analyzed by immunoblotting and qPCR. Both MCVmc.VP1-mS and MCVmc.VP1-P2A-mS express LT at comparable levels to MCVmc at days 2 and 4 post transfection (Figure 4B). As expected, VP1 (~50 kDa) shifted to higher molecular masses for MCVmc.VP1-mS and MCVmc.VP1-P2A-mS as a result of the residual fusion peptide/protein. DpnI-resistant DNA detection (Figure 4C) showed comparable increases in MCV genome copy numbers for MCVmc and the reporter viruses, although the mScarlet fusion protein virus (MCVmc.VP1-mS) had consistently reduced replication. Using a primary mouse anti-VP1 followed by a AF488-conjugated secondary anti-mouse antibody, dual color IF of transfected cells showed complete nuclear co-localization of mScarlet (red) with VP1 (green) for the MCVmc.VP1-mS fusion virus, (left panel, Figure 4D). In contrast, cells transfected with the MCVmc.VP1-P2A-mS virus showed mScarlet staining also in the cytoplasm, consistent with a cleaved fluorescent tag. Comparison of LT and mScarlet expression revealed discrete localization of LT protein at putative nuclear replication puncta (right panel, Figure 4D).

### 3.6. Replication Kinetics for MCV Reporter Viruses

**Figure 5:**
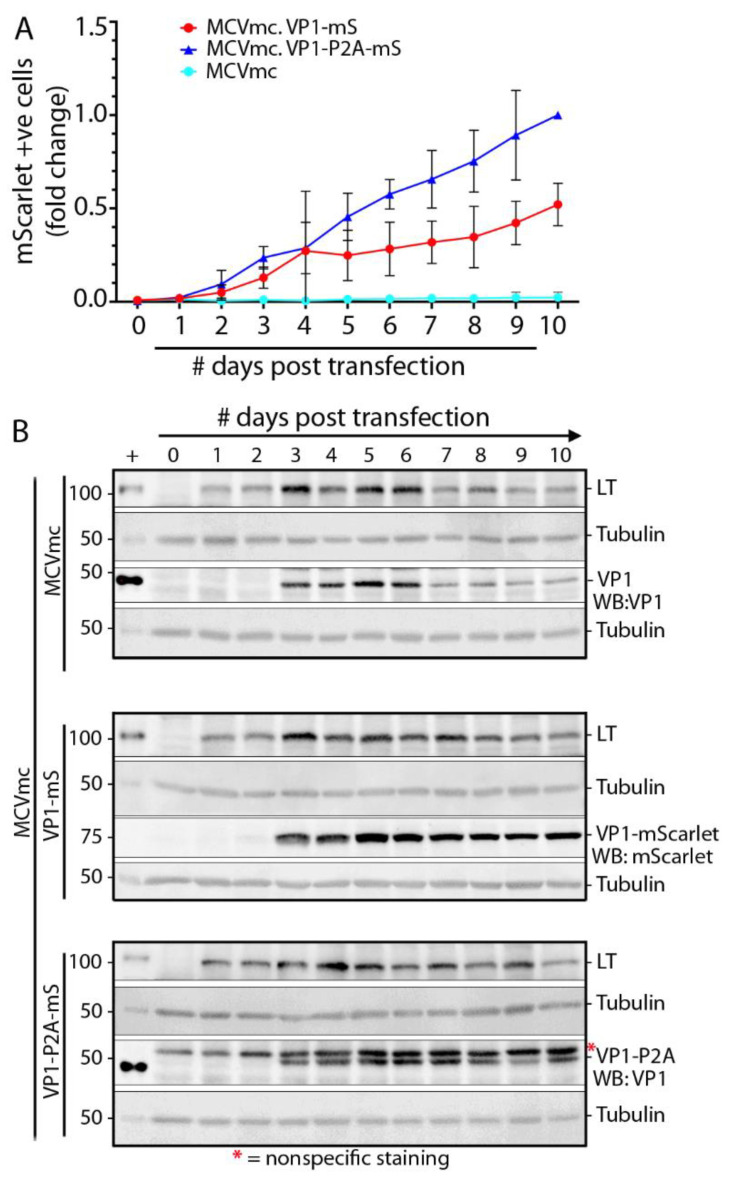
MCV replication kinetics analysis using mScarlet reporter. (**A**) Numbers of mScarlet-positive cells in 293 cells transfected with MCVmc, MCVmc.VP1-mS, or MCVmc VP1-P2A-mS from day 1 through day 10 post transfection were quantified by flow cytometry. Data were normalized to MCVmc.VP1-P2A-mS day 10 post transfection. Error bars indicate ± SD of three independent experiments. (**B**) Immunoblot of MCV-encoded proteins (LT and VP1) or mScarlet in 293 cells transfected with MCVmc, MCVmc.VP1-mS, and MCVmc. VP1-P2A-mS from 1 to 10 days post transfection. LT or VP1 expression construct-transfected 293 cells were used as a positive control (+); while α-tubulin was used as an endogenous protein-loading control. Protein molecular weight markers are shown on the left. Results are representative of three independent experiments. *—Indicates non-specific staining.

Since sT is involved in MCV replication, MCVmc, MCVmc.VP1-mS or MCVmc.VP1-P2A-mS were transfected into wild type 293 cells instead of 293 TRE-sT cells and monitored by flow cytometry and immunoblotting to analyze MCV replication kinetics (Figure 5A,B). mScarlet expression from both fusion viruses was evident as early as day 2 post transfection and the number of mScarlet-positive cells increased through 10 days post transfection. Consistent with data from Figure 4, MCVmc.VP-P2A-mS showed a growth advantage as measured by the number of positive cells compared to MCVmc.VP1-mS. All three viruses showed highest LT protein expression at days 3–6 which declined at later time points (days 8–10). VP1, in contrast, peaked at days 5–6 (Figure 5B).

### 3.7. Single-Round Transmission of MCV mScarlet Reporter Viruses

**Figure 6:**
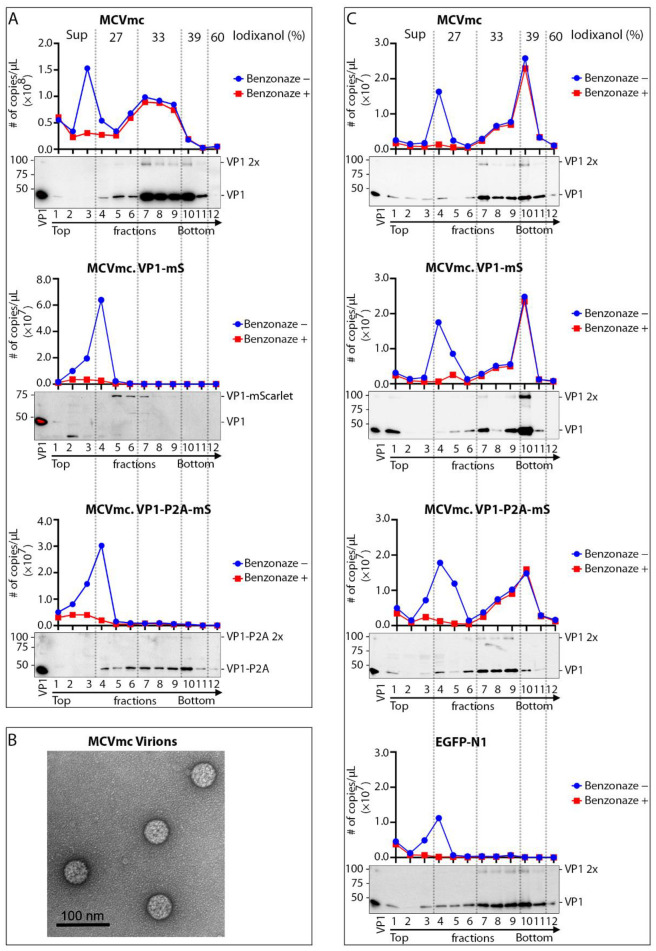
Virus production and infection using MCV mScarlet reporter virus. (**A**) Western blot and qPCR analysis of fractions from MCVmc, MCVmc.VP1-mS, and MCVmc.VP1-P2A-mS virions purified over an Opti-Prep gradient (Iodixanol) (concentrations noted on top). In the qPCR quantification, the blue curve shows total MCV DNA copy number, while the red curve shows Benzonase-protected MCV DNA copy numbers. Results represent two independent experiments. (**B**) Image of MCVmc virions (from (**A**)) by negative staining electron microscopy. (**C**) Western blot and qPCR analysis in Opti-Prep fractions from heterologous VP1/VP2 packaged MCVmc, MCVmc.VP1-mS, and MCVmc.VP1-P2A-mS. Results represent one-time experiments. Iodixanol concentration for each fraction is noted on top. qPCR quantification: blue curves show total MCV DNA copy number, while red curves show the number of Benzonase-resistant MCV DNA copies.

MCVmc.VP1-mS and MCVmc.VP1-P2A-mS DNAs were transfected into 293 cells, harvested after 10 days, lysed and purified on a 27–60% discontinuous OptiPrep gradient. Collected fractions were treated with Benzonase or left untreated before MCV DNA quantification by qPCR (Figure 6A). All preparations showed a peak of Benzonase-sensitive DNA at fractions 3–4, consistent with unencapsidated DNA in protein aggregates [54]. MCVmc.VP1 banding was present in fractions 7–10 that co-migrated with the Benzonase-protected MCV genome, consistent with full encapsidation. Neither MCVmc.VP1-mS nor MCVmc.VP1-P2A-mS generated Benzonase-protected MCV DNA. For MCVmc.VP1-P2A-mS, VP1 was present in dense fractions, consistent with multimerization without generation of complete capsid.

To generate encapsidated MCV mScarlet reporter viruses, q heterologous MCV genome was co-transfected together with expression plasmids for the native VP1 and VP2 proteins at a 3:1 ratio and tested for packaging efficiency in parallel with MCVmc.

**Figure 7:**
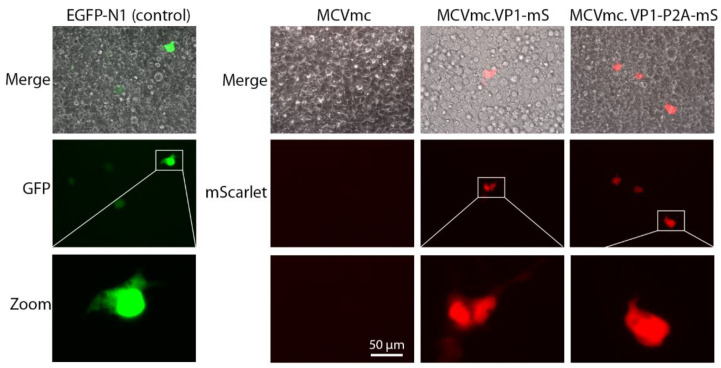
Single-round infection by pseudovirus-packaged MCV reporter. Images represent 293 TRE-sTco cells infected with exogenous VP1/VP2-packaged pEGFP-N1 (pseudo color green), MCVmc, MCVmc.VP1-mS (pseudo color red) or MCVmc.VP1-P2A-mS (red) reporter. Images were originally acquired at 40× magnification.

Complementation of VP1 and VP2 proteins in trans rescued encapsidation of both MCVmc.VP1-mS and MCVmc.VP1-P2A-mS genomes, as evidenced by the appearance of MCV genomic DNA in the VP1-enriched heavy fraction corresponding to encapsidated genomes (Figure 6C). Infection using 5 × 10^8^ genome copies (MOI 2 × 10^3^) in 293 TRE-sTco cells generated a small number of mScarlet-expressing cells 8–10 days after infection, representing a single round of infection using the pseudovirus-packaged fluorescent-encoded MCV genomes (MCVmc.VP1-mS: 57 cells/well; MCVmc.VP1-P2A-mS: 62 cells/well; and EGFP-N1 (control): 2063 cells/well) (Figure 7 and Figure S3).

## 4. Discussion

To gain a better understanding of MCV biology, we generated an MCVmc that enabled the production of a covalently closed circular genome free of bacterial sequences and amenable to genetic manipulation (Figure 1). Use of MCVmc improves experimental reproducibility due to the presence of only a single copy of the viral genome per minicircle in contrast to in vitro ligated MCV genomes (Figure 1A) that can exist as concatenated forms produced by in vitro ligation. In addition to the ease of minicircle production, we find that MCVmc replication and gene expression is similar to in vitro ligated MCV genomes, underscoring the validity of this system for studying the MCV lifecycle.

**Figure 8:**
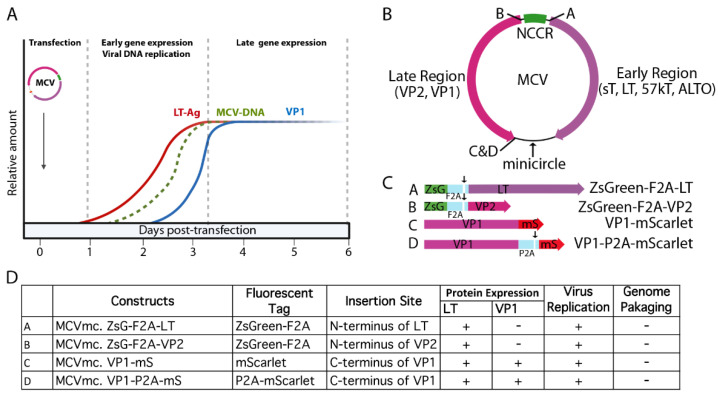
Kinetics of MCVmc reporter viruses. (**A**) A model of MCV replication kinetics, early/late gene expression as well as viral DNA replication is depicted. LT-Ag and VP1 expression kinetics is based on the Western blots in Figure 5. The MCV genome replication kinetics is hypothetical. (**B**) A map of MCVmcs with sites of fluorescent protein cassettes and minicircle vector insertion is indicated. (**C**) A detailed schematic of fluorescent-tagged MCV viral proteins that can be expressed from (**A**). Arrows in (**B**) indicate ribosome-skipping sites for F2A and P2A peptide sequences. (**D**) A table of tested MCVmc fluorescent reporter genomes. +: detected in transfected cells; −: not detected in transfected cells.

Minicircle technology has been used for stable gene expression and circularization of small DNA virus genomes, such as HBV and HPV free from bacterial sequences [48,49,50]. Limitations in the system described here derives from the fact that MCV is the smallest genome generated using this recombination approach. The constraints on placing recombination sites and fluorescent protein insertions into the MCV genome without eliminating viral viability required multiple trial and error cloning attempts (Figure 8). The recombination site leaves an extra 39 bp sequence and careful selection of its location was required to retain virus functionality. As shown in Figure 8, introduction of exogenous sequences into MCV genes can alter protein expression and genome packaging. While the recombinant MCV minicircle is a valuable new technology for defining MCV biology, careful comparisons are required to assess potential changes to the virus and its lifecycle caused by exogenous sequences. We do not find evidence that the recombination site we selected between the c-termini of the VP1 and LT genes and the “scar” of extraneous sequences affects virus replication or gene expression, but this remains a potential caveat to this approach. We find that introduction of the MCV350 NCCR mutation (Rep^–^) [11] eliminates late protein VP1 expression, consistent with newly replicated viral DNA being required for VP1 protein expression. The β-TrCP phosphorylation site at LT aa 149 is similarly required for replication and late gene, but not early gene, expression (Figure 3A). Conversely, introduction of mutations that abolished restriction factors for MCV replication, including LT-binding sites for Vam6p [41], interaction residues to SCF E3-ligases Fbw7 and Skp2 [12] or the MCV miR-M1 hairpin [15] markedly increase VP1 expression. Of note, some mutants displayed cell-type dependent effects. Whereas Fbw7 and hVam6p mutants show these host cell proteins to be replication restriction factors in 293 cells, they are neutral in U2OS cells (Figure 3). Different cell lines are characterized by different expression programs and profiles, which can greatly affect many biological processes. Similar to MCCs, 293 cells are of neuroendocrine origin, expressing neurofilaments and cytokeratin [55,56], whereas U2OS are of osteosarcoma origin. It is possible that variation in the expression of specific host cell proteins between these cell lines may contribute to the cell type-specific effects we observed.

Addressing practical implementation, introduction of a fluorescently tagged viral protein facilitates direct visualization of viral gene expression and and the determination of MCV replication kinetics (Figure 5 and Figure 8). Extensive manipulation shows that the site of fluorescent reporter placement is critical. While fusion of mScarlet (mS) to the VP1 c-terminus was tolerated for full genome replication and late gene expression, introduction of fluorescent tags (mS or ZsGreen, ZsG) on either side of the NCCR, in the sense (5′ of LT coding sequence) or antisense (5′ to VP2 coding sequence) orientation eliminated late gene expression (Figure 8B–D). This could be due to interference with proper late gene leader-to-leader splicing [51,52,57] or disruption of transcriptional regulatory elements in the NCCR. Ribosome-skipping sequences (P2A or foot-and-mouth disease virus ribosome skipping sequence, F2A: GSGVKQTNLFDLLKLAGDVESNPGP) cloned between VP1, VP2 or LT and fluorescent reporter peptides may minimize interference to replication when compared to their bulky fusion counterparts (Figure 4C). However, because the fluorescent reporter tags are cleaved from their corresponding, co-translated viral protein by ribosome skipping, actual fluorescent signals cannot serve as surrogates for localization purposes. Hence, the increased intensity of the cytoplasmic signal from MCVmc.VP1-P2A-mS compared with the predominantly nuclear signal in MCVmc.VP1-mS is likely to reflect cytoplasmic accumulation of cleaved reporter protein rather than VP1 (Figure 4D).

Development of the VP1 reporter virus allows measurement of replication kinetics after genome transfection by flow cytometry (Figure 5) which can also be applied to real-time live-cell microscopy. However, neither the VP1-mS nor VP1-P2A-mS minicircle virus was able to produce Benzonase-protected encapsidated virus genomes and no viral particles were visualized in heavy gradient fractions by electron microscopy. This is likely due to extra amino acids at the VP1 C-terminus (the P2A site leaves a c-terminal 21 amino acid “tag” on VP1) (Figure 4). Previous structure studies of MCV viral particles show that alterations at the VP1 c-terminus are functionally crucial. Deletions, including a 37 amino acid sequence unique to MCV, disrupted the ability of VP1 to form VLP [58]. Our studies clearly show that addition of even 21 aa onto the VP1 c-terminus disrupts virion assembly.

We used a protocol similar to that reported by Liu et al., [27] to infect a panel of primary cells. Infection with MCV-ligated [27,59] and MCVmc-produced virions that can in turn infect primary HFF-1 cells, BJ and immortalized BJ-hTert cells, while infection of human mesenchymal stem cells is minimal (Figure 2). Use of single-round infection with MCVmc.VP1-mS and MCVmc.VP1-P2A-mS reporter viruses (Figure 7), packaged into VLP and gradient-purified, can allow the ready manipulation of factors regulating MCV entry, uncoating and genome replication [18,25,26].

MCVmc provides an easy and efficient tool to assay MCV replication and its interaction with the host. Our studies confirm the roles of restriction factors in controlling MCV replication, including the Fbw7 interaction site in the LT protein. Fluorescent gene expression coupled with late gene expression provides an excellent tool to visualize MCV replication kinetics in real time and for use in high-throughput screening targeting MCV replication.

## Figures and Tables

**Table 1:** Construction summary of pMC(minicircle)-MCV (Merkel cell polyomavirus) harboring mutations.

Final Constructs	Mutation Sites	Insert Source	Restriction Enzymes
pMC-MCV-Rep^–^	C44A	pJ-MCV-HF-Rep^–^	*Avr*II, *Sac*I
pMC-MCV-hVam6p^–^	T1251G, G1252C	pJ-MCV-HF-hVam6p^–^	*Bam*HI, *Eco*RI
pMC-MCV-β-TrCP^–^	T1065G	pJ-MCV-HF-β-TrCP^–^	*Avr*II, *Bam*HI
pMC-MCV-Skp2^–^	T1284G	pJ-MCV-HF-Skp2^–^	*Bam*HI, *Eco*RI
pMC-MCV-Fbw7^–^	T1341G	pJ-MCV-HF-Fbw7^–^	*Bam*HI, *Eco*RI

**Table 2:** List of primers used for overlapping PCR and Gibson assembly.

VP1 (2440-2466) F	TGACACATTGCAGATGTGGGAGGCAAT
VP1-mScarlet R	GCCTCGCCCTTGCTCACCATTAATTCTTGTGTTTGGCTTT
VP1-mScarlet F	AAAGCCAAACACAAGAATTAATGGTGAGCAAGGGCGAGGC
mScarlet-attBVec R	TCCCCGGGCGCGACAAATAATTCTCACTTGTACAGCTCGT
VP1-P2A R	GTCTCCAGCCTGCTTCAGCAGGCTGAAGTTAGTAGCTCCGCTTCCTAATTCTTGTGTTTGGCTTTCTTTTTGAGAGGCC
P2A-mScarlet F	AGCCTGCTGAAGCAGGCTGGAGACGTGGAGGAGAACCCTGGACCTATGGTGAGCAAGGGCGAGGCA
VP1-P2A_FW	GCCAAGCTTGCATGCCGTTCTTTAATTAATGTTCATTATT
VP1-P2A_RV	CCAGCCTGCTTCAGCAGGCTGAAGTTAGTAGC
P2A-mScarlet_FW	TGCTGAAGCAGGCTGGAGACGTGGAGGAGAAC
P2A-mScarlet_RV	AATTCGAGCTCGGTACTCCCCGGGCGCGACAAATAATTCT

## Data Availability

All relevant data are within the manuscript and its Supplementary Materials files.

## Supplementary Materials

The following supporting information can be downloaded at: https://www.mdpi.com/article/10.3390/v14030473/s1, Figure S1: Infection of Primary Cells. Figure S2: Viral genome mutational analysis by using MCVmc system. Figure S3: Quantification of single round infection by pseudovirus packaged MCV reporter. Table S1: List and description of plasmid constructs.
