# Supplementary material for: Replication Kinetics for a Reporter Merkel Cell Polyomavirus
Source: Viruses. 2022 Feb 25;14(3):473. doi: 10.3390/v14030473 (PMC8950423; doi:10.3390/v14030473)
Supplement: Supplementary file 1 [file viruses-14-00473-s001.zip › viruses-1607792-supplementary.pdf]

viruses

Supplementary Materials

Replication Kinetics for a Reporter Merkel Cell Polyomavirus

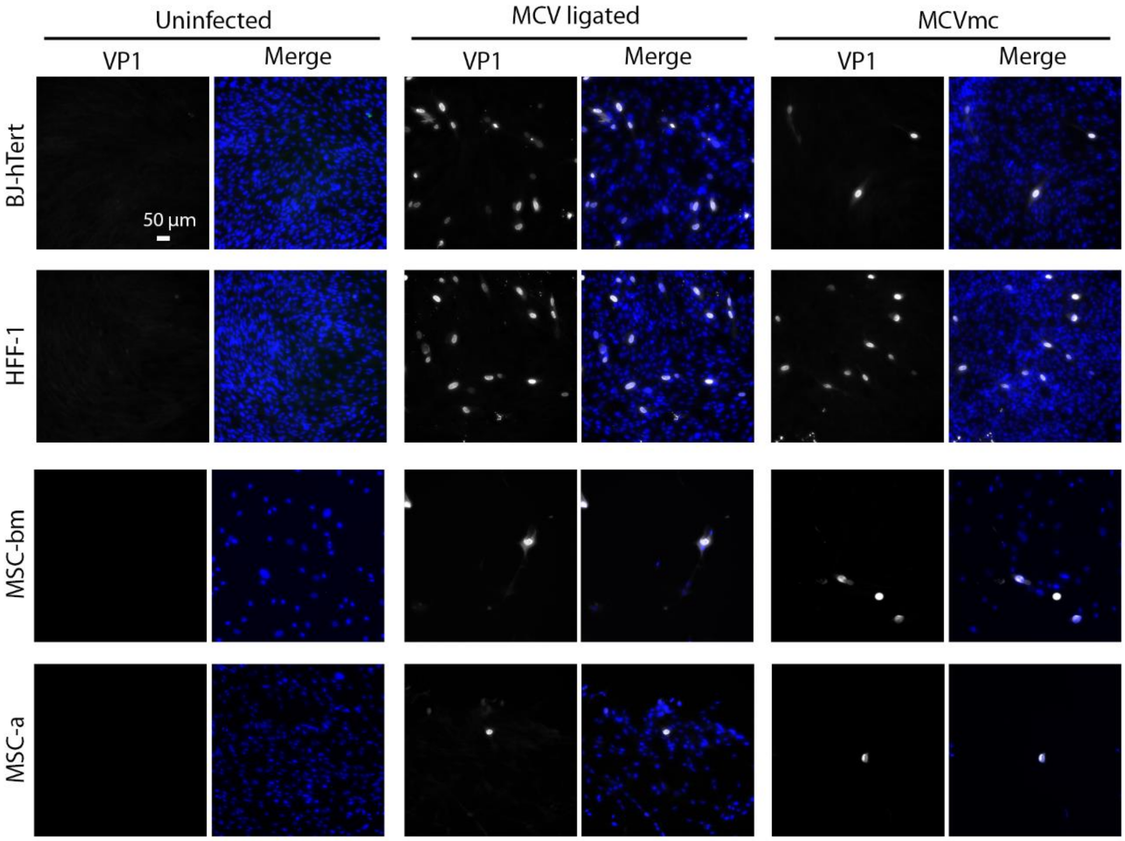

Figure S1. Infection of Primary Cells. Immunofluorescence of VP1-AF488 (white) in MCV ligated and MCVmc infected BJ-hTert, HFF-1, MSC-bm, or MSC-a cells 6-day post-infection. Images were originally acquired at 40× magnification. DAPI counter staining is shown in blue.

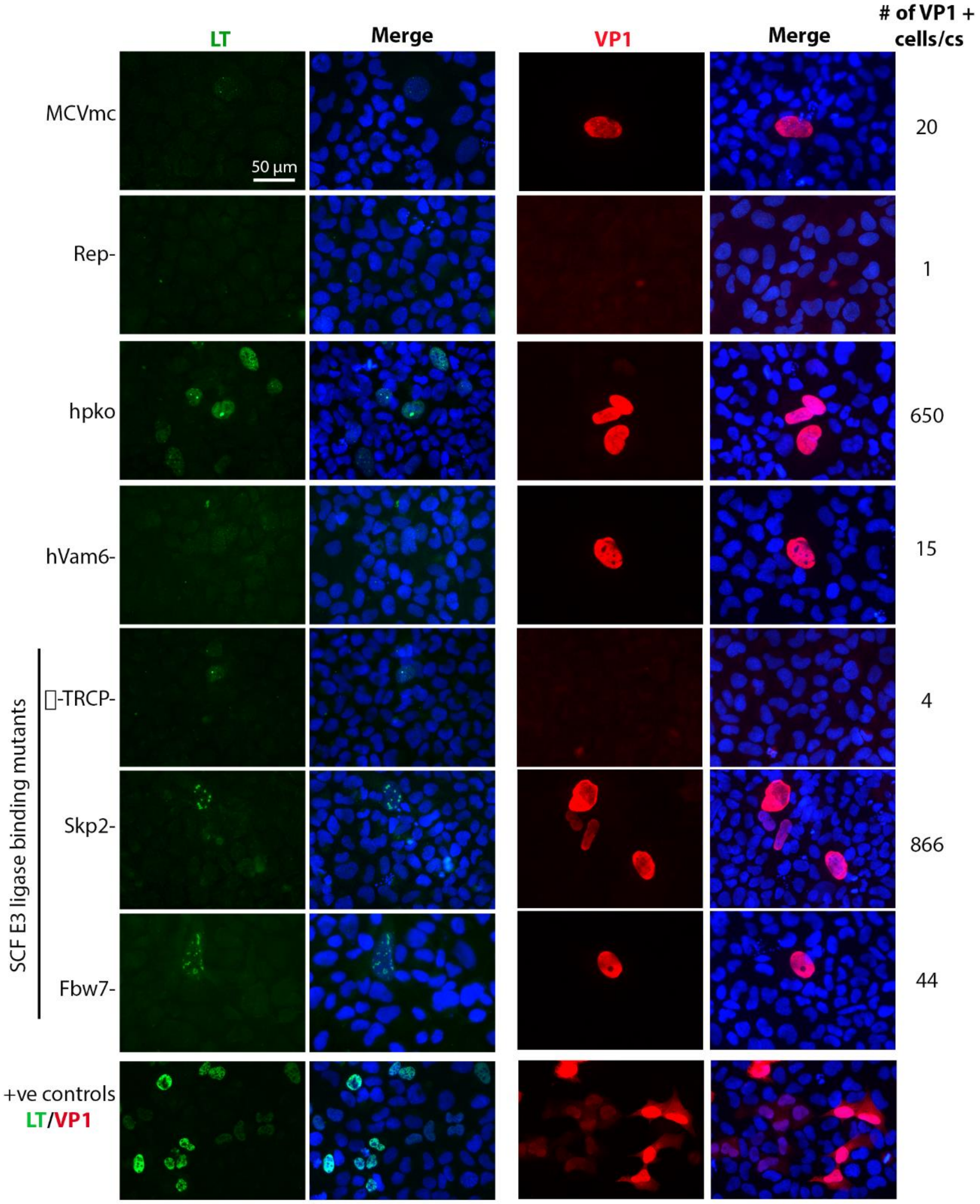

Figure S2. Viral genome mutational analysis by using MCVmc system. Immunofluorescence analysis of LT-AF488 (pseudo color green) or VP1-AF488 (pseudo color red) in U2OS cells transfected with MCVmc or mutants 5-day post-transfection. LT and VP1 expression construct-transfected U2OS cells were used as positive controls. Images were originally acquired 40× magnification. The number of VP1 positive cell on each coverslip was quantified by using Cytation 5 cell imaging multimode reader.

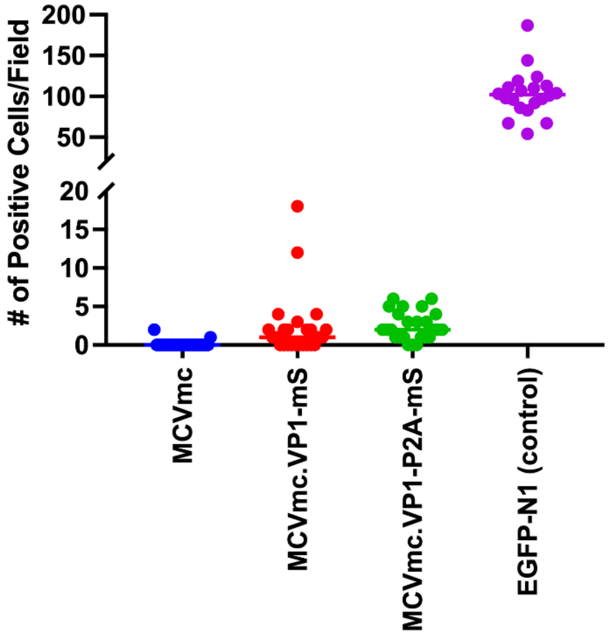

Figure S3. Quantification of single round infection by pseudovirus packaged MCV reporter. The number of mScarlet or EGFP positive cell were counted using Cytation 5 cell imaging multi-mode reader. Each dot represents the numbers of mScarlet or EGFP positive 293 cells per field 10 days post-infection.

Table S1. List and description of plasmid constructs.

Construct	Function	Parental Vector	Chang-Moore Plasmid #	ChangMoore Plasmid Name	Cat#
pSMART-MCV-HF	Amplifies MCV-HF genome in bacteria for MCV genome re-circularization by re-ligation after enzyme digestion which release MCV-HF genome from bacterial backbone.	pSMART	4556	MCV-HF	N/A
pJ-MCV-Rep⁻	The donor of Rep- mutation (C44A) for minicircle cloning.	pJ	3148	MCV-Rep⁻	N/A
pJ-MCV-hVam6p⁻	The donor of LT hVam6p binding deficent mutation (T1251G, G1252C) for minicircle cloning.	pJ	3149	MCV-hVam6p⁻	N/A
pJ-MCV-β-TrCP⁻	The donor of LT β-TrCP binding deficent mutation (T1065G) for minicircle cloning.	pJ	3987	MCV-HF/LT.S147A	N/A
pJ-MCV-Skp2⁻	The donor of LT Skp2 binding deficent mutation (T1284G) for minicircle cloning.	pJ	3988	MCV-HF/LT.S220A	N/A

pJ-MCV-Fbw7⁻	The donor of LT Fbw7 binding deficent mutation (T1341G) for minicircle cloning.	pJ	3989	MCV-HF/LT.S239A	N/A
pMC-MCV	Amplifies MCV-HF genome in bacteria for MCV genome re-circularization by recombination.	pMC.BE-SPX	4587	pMC.BESPX-MCV-HF	N/A
pMC-MCV-Rep⁻	Amplifies replication deficient MCV-HF genome in bacteria for MCV genome re-circularization by recombination.	pMC.BE-SPX	4672	pMC.BESPX-MCV-HF-Rep⁻	N/A
pMC-MCV-hVam6p⁻	Amplifies MCV-HF genome that expresses hVam6p binding deficient LT in bacteria for MCV genome re-circularization by recombination.	pMC.BE-SPX	4673	pMC.BESPX-MCV-HF-hVam6p⁻	N/A
pMC-MCV-β-TrCP⁻	Amplifies MCV-HF genome that expresses β-TrCP binding deficient LT in bacteria for MCV genome re-circularization by recombination.	pMC.BE-SPX	4674	pMC.BESPX-MCV-HF-β-TrCP⁻	N/A
pMC-MCV-Skp2⁻	Amplifies MCV-HF genome that expresses Skp2 binding deficient LT in bacteria for MCV genome re-circularization by recombination.	pMC.BE-SPX	4675	pMC.BESPX-MCV-HF-Skp2⁻	N/A
pMC-MCV-Fbw7⁻	Amplifies MCV-HF genome that expresses Fbw7 binding deficient LT in bacteria for MCV genome re-circularization by recombination.	pMC.BE-SPX	4676	pMC.BESPX-MCV-HF-Fbw7⁻	N/A
pMC-MCV-VP1-mScarlet	Amplifies MCV-HF genome that expresses mScarlet-fused VP1 in bacteria for MCV genome re-circularization by recombination.	pMC.BE-SPX	4764	pMC.BESPX-MCV-HF-mScarlet	N/A
pMC-MCV-VP1-P2A-mScarlet	Amplifies MCV-HF genome that expresses individual mScarlet and VP1 using a P2A sequence in bacteria for	pMC.BE-SPX	4765	pMC.BESPX-MCV-HF-P2A-mScarlet	N/A

	MCV genome re-circularization by recombination.				
pwM	Expresses MCV VP1 protein in mammalian cells.	pGwf	2972	MCV VP1 (PWM)	Addgene# 22515
ph2m	Expresses MCV VP2 protein in mammalian cells.	phGf	2973	MCV VP2 (PH2M)	Addgene# 22518
pmScarlet-C1	Express mScarlet in mammalian cells.	pC1	4738	pmScarlet-C1	Addgene# 85042
pEGFP-N1	Express EGFP in mammalian cells.	pEGFP-N1	2437	pEGFP-N1	Clontech# 6085-1
